# Universal Oriented van der Waals Epitaxy of 1D Cyanide Chains on Hexagonal 2D Crystals

**DOI:** 10.1002/advs.201900757

**Published:** 2019-12-19

**Authors:** Yangjin Lee, Jahyun Koo, Sol Lee, Jun‐Yeong Yoon, Kangwon Kim, Myeongjin Jang, Jeongsu Jang, Jeongheon Choe, Bao‐Wen Li, Chinh Tam Le, Farman Ullah, Yong Soo Kim, Jun Yeon Hwang, Won Chul Lee, Rodney S. Ruoff, Hyeonsik Cheong, Jinwoo Cheon, Hoonkyung Lee, Kwanpyo Kim

**Affiliations:** ^1^ Department of Physics Yonsei University Seoul 03722 Korea; ^2^ Center for Nanomedicine Institute for Basic Science (IBS) Seoul 03722 Korea; ^3^ Department of Physics Konkuk University Seoul 05029 Korea; ^4^ Department of Physics Sogang University Seoul 04107 Korea; ^5^ Department of Physics Ulsan National Institute of Science and Technology (UNIST) Ulsan 44919 Korea; ^6^ Center for Multidimensional Carbon Materials (CMCM) Institute for Basic Science (IBS) Ulsan 44919 Korea; ^7^ Department of Physics and Energy Harvest‐Storage Research Center University of Ulsan Ulsan 44610 Korea; ^8^ Institute of Advanced Composite Materials Korea Institute of Science and Technology (KIST) Jeonbuk 55324 Korea; ^9^ Department of Mechanical Engineering Hanyang University Ansan 15588 Korea; ^10^ Department of Chemistry Ulsan National Institute of Science and Technology (UNIST) Ulsan 44919 Korea; ^11^ School of Materials Science and Engineering Ulsan National Institute of Science and Technology (UNIST) Ulsan 44919 Korea; ^12^ School of Energy and Chemical Engineering Ulsan National Institute of Science and Technology (UNIST) Ulsan 44919 Korea; ^13^ Department of Chemistry Yonsei University Seoul 03722 Korea; ^14^ Graduate Program of Nano Biomedical Engineering Yonsei‐IBS Institute Yonsei University Seoul 03722 Korea

**Keywords:** 1D cyanide chains, 2D hexagonal crystals, oriented van der Waals epitaxy, vertical heterostructures

## Abstract

The atomic or molecular assembly on 2D materials through the relatively weak van der Waals interaction is quite different from the conventional heteroepitaxy and may result in unique growth behaviors. Here, it is shown that straight 1D cyanide chains display universal epitaxy on hexagonal 2D materials. A universal oriented assembly of cyanide crystals (AgCN, AuCN, and Cu_0.5_Au_0.5_CN) is observed, where the chains are aligned along the three zigzag lattice directions of various 2D hexagonal crystals (graphene, h‐BN, WS_2_, MoS_2_, WSe_2_, MoSe_2_, and MoTe_2_). The potential energy landscape of the hexagonal lattice induces this preferred alignment of 1D chains along the zigzag lattice directions, regardless of the lattice parameter and surface elements as demonstrated by first‐principles calculations and parameterized surface potential calculations. Furthermore, the oriented microwires can serve as crystal orientation markers, and stacking‐angle‐controlled vertical 2D heterostructures are successfully fabricated by using them as markers. The oriented van der Waals epitaxy can be generalized to any hexagonal 2D crystals and will serve as a unique growth process to form crystals with orientations along the zigzag directions by epitaxy.

## Graphical abstract

1D cyanide chains show universal oriented van der Waals epitaxy on various hexagonal 2D crystals. The chain directions are always aligned along the zigzag lattice directions of the 2D substrates, regardless of differing lattice parameters and surface elements. Stacking‐angle‐controlled vertical 2D heterostructures can also be fabricated using cyanide microwire markers for identification of crystal orientations.

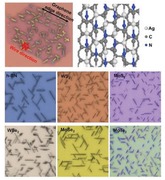

Epitaxial growth is a key process for the realization of high‐quality thin‐film crystals and nanostructures on various substrates.^1^ The crystal formation from adsorbed atoms on a substrate can depend on a variety of parameters, including particularly the competition between atom–atom and atom–substrate interactions.^1^ Previous studies on heteroepitaxy have demonstrated that the lattice matching and chemical interaction between the substrate and deposited material are very important, which mainly govern the quality, growth mode, and degree of strain in the deposited structure. Therefore, high‐quality epitaxy of a certain material can be realized only when a substrate is properly chosen, based on its lattice parameters and chemical interactions.

Recently, emerging 2D materials have been investigated for their novel phenomena and potential applications in various electronics.^2^ One interesting aspect of this family of materials is their use as a substrate for assembling other functional materials.^3^ The assembly process on some of these 2D materials is mainly driven via a relatively weak van der Waals interaction, which is quite distinct from conventional heteroepitaxy. Previous studies have demonstrated that molecules and polymers can organize themselves to well‐ordered structures on various 2D crystals.^3^ However, these studies only focused on individual assembly cases, and obtaining a general epitaxial mechanism over various 2D crystals has been challenging. The recently observed “remote epitaxy” through single‐layer graphene suggests that the material growth mediated by a relatively weak van der Waals force may host unexpected epitaxial behavior.^4^

We demonstrate that 1D cyanide chains show universal oriented epitaxy on various hexagonal 2D crystals, regardless of differing lattice parameters and surface elements. AgCN microwires, close‐packed structures of AgCN chains,^5^ were found to form on various hexagonal 2D crystals (graphene, h‐BN, WS_2_, MoS_2_, WSe_2_, MoSe_2_, and MoTe_2_), displaying universal threefold rotational epitaxy. By analysis of transmission electron microscopy (TEM) images and selected area electron diffraction (SAED) patterns, we found that the cyanide chain directions are always aligned along the zigzag lattice direction of these substrates. Other cyanide chains (AuCN and Cu_0.5_Au_0.5_CN) also showed the same oriented epitaxial behavior, suggesting the existence of a universal mechanism for such alignment. Using first‐principles calculations and the surface potential for molecular adsorption, we found that the potential landscape of the hexagonal lattice induces the alignment of 1D chains along the zigzag lattice directions, regardless of the lattice parameter and the degree of surface interactions. We also fabricated, with fine control of the stacking angle, vertical 2D heterostructures using microwire markers to identify crystal orientation. Oriented van der Waals epitaxy can be useful for manipulating the growth of materials as well as to readily determine the crystal directions of various 2D materials.

Single‐crystalline AgCN microwires can be deposited on various 2D crystals by simple drop‐casting of a AgCN solution. A solution of AgCN in concentrated ammonia was dropped onto a 2D crystal/SiO_2_/Si substrate and the samples were dried under mild heating on a hot plate in air, which resulted in the formation of AgCN microwires on 2D crystals, as shown in **Figure** **1**a (see the Experimental Section for detailed information). Single‐layer and single‐crystal graphene island samples were used to study the growth phenomena of AgCN as shown in Figure 1b. Optical microscope images clearly showed that the microwires were oriented with threefold symmetry on graphene, which suggested that the hexagonal symmetry of graphene guides the epitaxial alignment of AgCN crystals (Figure 1b). The dimensions of the AgCN microwires on graphene were ≈1 µm, ≈3 µm, and ≈300 nm (width, length, and height, respectively; Figure S1, Supporting Information), and can be tuned by controlling the growth conditions. The use of hexagonal single‐crystal graphene islands (Figure S2, Supporting Information) allowed for the study of the wire axis direction relative to the graphene crystal orientation. Previous studies have shown that the edges of the hexagonal graphene domains are along the zigzag lattice direction.^6^ The wire axis was parallel to the graphene crystal edges (Figure 1b) from the optical microscope images, and this suggested that the wire axis was predominantly aligned along the zigzag lattice direction of graphene.

**Figure 1:**
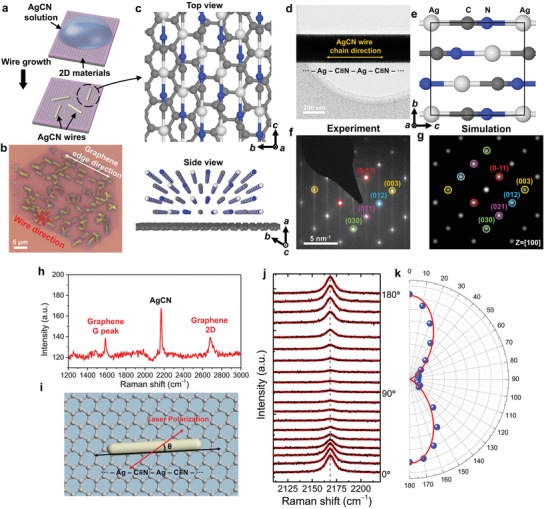
Van der Waals epitaxy of AgCN chains on graphene. a) Schematic diagram of the microwire growth process. b) Optical image of the AgCN wires on a single‐domain graphene flake. c) Atomic configuration of the AgCN wire and graphene: top (upper) and side view from the wire axis direction (bottom). The white, gray, and blue spheres represent Ag, C, and N atoms, respectively. d) TEM image of an AgCN microwire. e) Crystal structure of AgCN on the [100] zone axis. f) Experimental SAED pattern of the AgCN wire in (d). g) Simulated electron diffraction of AgCN on the [100] zone axis. h) Raman spectra of a AgCN wire on graphene. i) Schematic illustration of the Raman spectroscopy with polarized excitation. θ is the angle between the wire axis and laser polarization direction (parallel polarization, 0^°^ denotes the direction parallel to the wire chain direction). j) Polarized Raman spectra of the AgCN wires on graphene over the range 2100–2250 cm^−1^. The red curves represent the Lorentzian fitting results. k) Polar plots of the ν(C≡N) Raman intensity versus polarization angle. The red solid curve is a cos^4^θ fit to the data.

The AgCN microwires, in particular the direction of the cyanide chain axis, were characterized by various tools including TEM imaging, SAED, and polarized Raman spectroscopy. We performed TEM imaging and SAED of the AgCN wires (Figure 1d,f). The observed SAED (Figure 1f) agreed well with the simulated diffraction of the AgCN crystal structure for the [100] zone axis (Figure 1e,g). By using real‐space imaging and rotation‐calibrated SAED, the microwire axis was observed to be along the cyanide chain direction (Figure 1d,e). X‐ray photoemission spectroscopy confirmed the presence of silver and nitrogen in the AgCN microwires (Figure S3, Supporting Information).

Polarized Raman spectroscopy was used to evaluate the 1D vibrational properties of the AgCN chains. Figure 1h shows a typical Raman spectrum of AgCN wires grown on graphene. The peak near 2167 cm^−1^ is from the C≡N vibration mode.^7^ We measured the Raman spectra of the AgCN wires as a function of incident laser polarization angle, θ, with respect to the wire axis (Figure 1i). We observed a strong polarization‐dependent Raman peak intensity, ν(C≡N), as shown in Figure 1j. The maximum intensity of ν(C≡N) was observed when the laser polarization direction was parallel to the wire axis (at 0° and 180°). The polar intensity plot of the C≡N mode was well fitted to the cos^4^θ function (Figure 1k), which strongly indicated a 1D‐like vibrational mode of AgCN chains in the crystal.^8^

The oriented epitaxy of AgCN on graphene suggested exploring other hexagonal 2D crystals as shown in **Figure** **2**. Figure 2a–f shows optical microscope images of AgCN microwires grown on other hexagonal 2D crystals (h‐BN, WS_2_, MoS_2_, WSe_2_, MoSe_2_, and MoTe_2_), which show that the wire axis follows the threefold symmetry of the underlying hexagonal crystals. We caution that the optical microscope images cannot determine the direction of the crystal orientation along which the formation of the wire axis is preferred, although a single‐layer chemical vapor deposition (CVD) sample with well‐characterized edge termination (Figure S4, Supporting Information; the MoSe_2_ case) can be used to confirm the crystal direction, as also demonstrated for graphene. The morphology and density of wires grown on 2D substrates shows some hint of substrate‐dependence as shown in Figure 2a–f and Figure S5 in the Supporting Information. The observed substrate‐dependence may be originated from difference in degree of interaction between AgCN chains and various 2D substrates. However, we find that the morphology of wires often depends on the size and densities of exfoliated 2D flakes on SiO_2_/Si substrate. The uniform coverage of 2D samples using large‐area single‐crystal samples for growing wires may be necessary to clearly observe the substrate‐dependent morphological changes of wires.

**Figure 2:**
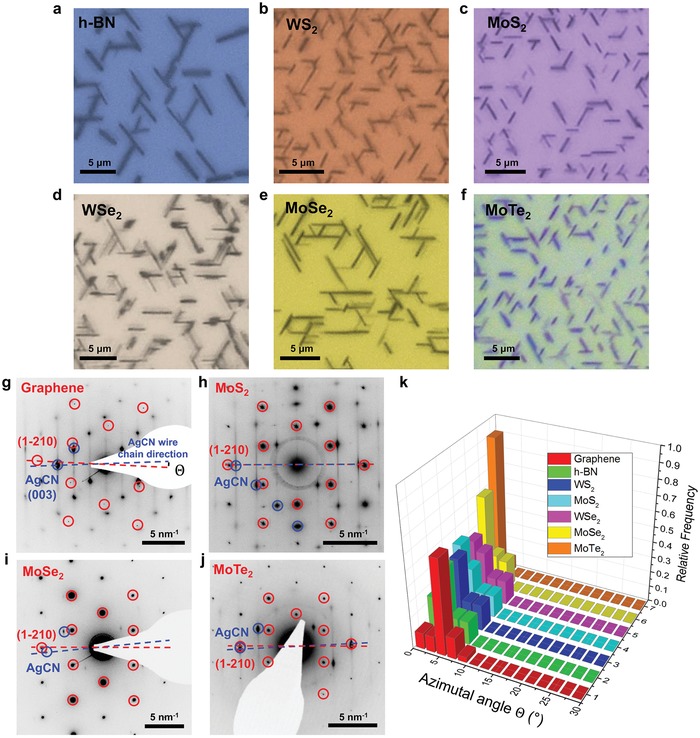
Universal oriented alignment of AgCN wires on various hexagonal 2D materials. a–f) Optical images of the AgCN wires on h‐BN, WS_2_, MoS_2_, WSe_2_, MoSe_2_, and MoTe_2_. SAED of the AgCN on g) graphene, h) MoS_2_, i) MoSe_2_, and j) MoTe_2_. k) Histogram of the distribution of angles of AgCN wires on various 2D materials as obtained by SAED.

To evaluate the crystal orientation of various 2D crystals and a possible preferred wire axis formation, we did SAED for AgCN/2D crystal samples. Figure 2g–j shows SAED patterns obtained from AgCN crystals grown on 2D crystals; more than 300 SAED were obtained and evaluated. (See Figure S6 in the Supporting Information for additional SAED data.) The azimuthal angle, *Θ*, between the (003) peak of AgCN and the (1‐210) peak of the 2D crystals was used to precisely analyze the azimuthal angles of the microwires. Figure 2k shows the histogram of *Θ*; the dominant population was ≈ 0° (zigzag lattice direction) for all of the hexagonal 2D crystals. The investigated hexagonal 2D crystals had different lattice parameters and elemental composition (see Table S1 in the Supporting Information). The SAED data demonstrates that the oriented epitaxy of 1D chains on hexagonal 2D crystals is a general phenomenon, regardless of the lattice parameters or chemical elements present in the underlying 2D crystals. We note that the azimuthal distribution of the wire axis showed a slight dependence on lattice parameter. The narrowest distribution was observed at 0° for MoTe_2_, where the lattice parameters of AgCN and MoTe_2_ produced the perfectly matched superstructure, 2 × *c*(AgCN) ≅ 3 × *A*(MoTe_2_) = 10.5 Å, as seen from the overlapped diffraction between the (003) AgCN and (1‐210) MoTe_2_ peaks (Figure 2j). High‐resolution TEM imaging can also be used to directly image the alignment behavior of AgCN chains on 2D crystals. TEM images shown in Figure S7 in the Supporting Information confirmed that the axis of AgCN chains is aligned along the graphene zigzag lattice direction.

The alignment of the chains was also observed for other cyanides (AuCN and Cu_0.5_Au_0.5_CN), which share similar atomic chain structures.^5^, ^7^ Our previous studies showed that AuCN chains exhibit similar oriented alignment behaviors on graphene, although the assembly of the chains resulted in nanowires, rather than micrometer sized crystals.^9^ A similar alignment of AuCN nanowires was also observed on other hexagonal 2D crystals, as shown in Figure S8 in the Supporting Information. Cu_0.5_Au_0.5_CN also showed a similar oriented van der Waals epitaxy and formed nanowires on various 2D crystals (Figure S9, Supporting Information). High‐resolution TEM imaging can also be used to directly image the alignment behaviors of Cu_0.5_Au_0.5_CN chains on graphene as shown in Figure S10 in the Supporting Information. This family of cyanides share a chain structure but have slightly different lattice parameters along the chain direction (Table S2, Supporting Information), which also supports our claim that the observed oriented van der Waals epitaxy of 1D chains is a universal behavior.

To try to understand the alignment of 1D chains on 2D hexagonal crystals, theoretical calculations and model analyses were used. First, we did first‐principles calculations of AgCN chains on MoS_2_ as a function of Θ between the three AgCN chains and the zigzag lattice direction of MoS_2_ (**Figure** **3**a). We found that the AgCN chains aligned along the zigzag lattice direction were favored (Figure 3b), which suggests that the oriented alignment behavior results from thermodynamic interaction between chains and hexagonal 2D crystals. We constructed the AgCN Lennard–Jones (LJ) potential on graphene and MoS_2_ using parameters obtained from first‐principles calculations (see the Experimental Section for detailed information). The LJ potentials between AgCN and 2D crystals were well fitted to the energy curves obtained from first‐principles calculations and our analysis can be easily extended to other 2D crystals. The potential energy maps of AgCN on graphene and MoS_2_ show that the energetically favorable locations (blue) for AgCN adsorption were different for graphene (at the center of the hexagon) and MoS_2_ (atop the transition metal atom), as indicated by the reversed color of the potential energy maps (Figure 3c,d). Even though the adsorption potential maps were different for AgCN/graphene and AgCN/MoS_2_, we found that the energetically favorable orientation of the chains was always along the zigzag lattice direction of the hexagonal crystals because of the hexagonal symmetry (Figure 3e,f; Figure S11, Supporting Information). From our observation, the oriented alignment behavior of cyanide chains does not depend on the size of assembled cyanide crystals. Chains that initially align along the zigzag direction can promote further growth of aligned nanowires, although the energetic difference between configurations of different azimuthal angles decreased as the size of a nanowire increased due to the lattice mismatching between the chains and 2D crystal.

**Figure 3:**
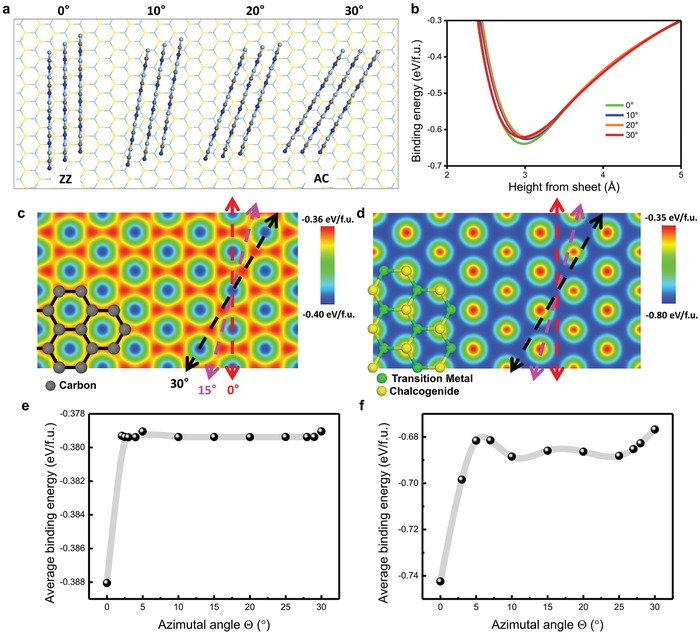
The alignment of 1D cyanide chains in certain directions on 2D hexagonal crystals. a) Atomic model of the AgCN wires on MoS_2_ with different azimuthal alignments. b) First‐principles calculations of the binding energy per AgCN functional unit (f.u.) on MoS_2_. 2D surface potential map of AgCN on c) graphene and d) MoS_2_. The blue color indicates the preferred adsorption position. The average binding energy of AgCN on e) graphene and f) MoS_2_ as a function of azimuthal angle.

The observed universal oriented epitaxy of AgCN microwires provides a useful way to determine the 2D crystal orientation and can be used to fabricate vertical 2D heterostructures with fine control of the twist angle between neighboring layers in the stack. Vertical 2D structures have received significant interest due to their unique physical properties and new functionality.[qv: 2c,10] In particular, the twist angle between 2D layers has been found to be one of the key parameters that largely control some important system properties and it is therefore useful to establish a simple way to control the twist angle for vertical heterostructures.[qv: 10e–i] Even though the bottom‐up synthesis of vertical 2D heterostructures has been realized for certain combinations of 2D materials,^11^ it is still challenging to find the optimal conditions for growth and to control the twist angle between 2D crystals. The alternative mechanical transfer/stacking of 2D materials for fabrication of vertical heterostructures requires knowledge of the crystal orientation of individual crystals. Previous methods for the identification of 2D crystal orientations include atomic resolution imaging by scanning tunneling microscope or TEM, second‐harmonic generation, and using the well‐defined edge of triangle or hexagonal‐shaped synthesized flakes.^12^ However, these methods require sophisticated measurement tools or bottom‐up synthesis of 2D crystals, and have limitations for widespread adoption and use for the identification of 2D crystal orientation.

**Figure** **4**a shows a schematic illustration of the overall fabrication process of vertical heterostructures with control of the stacking angle. The AgCN microwires are used as alignment markers for the stacking process. For demonstration purposes, we selected graphene and h‐BN for the vertical heterostructure.[qv: 10e,13] The AgCN wires were grown on the confined area of the 2D crystals, by protecting part of the 2D crystal flakes using polydimethylsiloxane, and the partly pristine 2D crystals for heterostructure fabrication were obtained. The rotational distribution of the microwires was analyzed to identify the crystal orientation (Figure S12, Supporting Information) and graphene/h‐BN vertical stacking was performed under the optical microscope with the aid of a micromanipulator. The flakes with zero twist angle between graphene and h‐BN were so stacked. The 2D heterostructure samples were transferred to TEM grids and analyzed using TEM.

**Figure 4:**
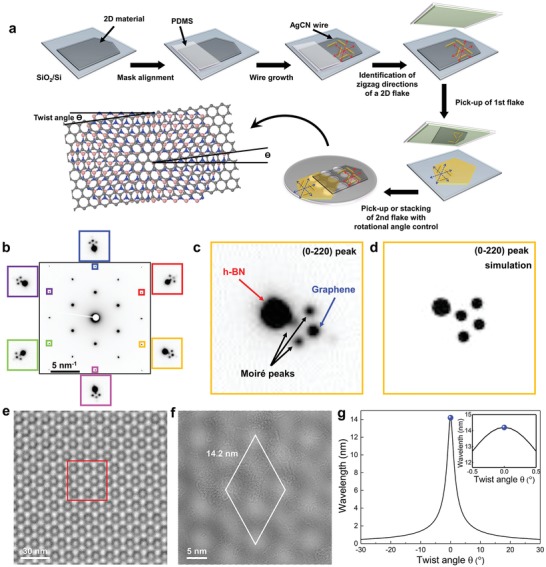
Fabrication of vertical heterostructures with stacking angle control. a) Schematic illustration of the overall process for fabrication of vertical 2D heterostructures with control of the stacking angle. b) SAED of graphene/h‐BN heterostructure with zero twist angle. The diffraction spots in the six color boxes are the zoomed‐in diffraction signals inside the black center box. c) Zoomed‐in diffraction signal in one of the boxes in panel (b). d) Simulated diffraction signal from graphene/h‐BN with zero twist angle. e) Phase‐contrast TEM image of the h‐BN/graphene heterostructure. f) Zoomed‐in image of the red box in (e). g) Moiré spacing as a function of twist angle between graphene and h‐BN. Inset: the moiré spacing over the range −0.5 to 0.5 twist angle. The blue dot indicates the experimentally measured moiré distance.

Figure 4b shows a SAED of the fabricated vertical heterostructure, which shows the zero twist angle between graphene and h‐BN. The diffraction spots from graphene and h‐BN strongly overlapped due to the small lattice mismatch. For relatively high index diffraction spots, h‐BN and graphene signals were distinguished, as shown in Figure 4b. The h‐BN and graphene (0‐220) diffraction spots were identified, as shown in Figure 4c, and the lattice parameter difference (1.76 ± 0.01%) and twist angle (<0.1^°^) were measured. We also observed moiré peaks in addition to the h‐BN and graphene diffraction spots. The simulated diffraction signals from the h‐BN/graphene heterostructure with zero twist angle reproduced the observed diffraction pattern (Figure 4d). The h‐BN/graphene moiré pattern was also directly observed using phase‐contrast TEM imaging (Figure 4e,f) and the observed moiré periodicity was consistent with the calculated value from the twist angle and the measured lattice parameter difference (Figure 4g). The moiré periodicity was found to be uniform over the observed region, which strongly suggests that the 2D interface between graphene and h‐BN was pristine. These results demonstrate that the microwire‐assisted identification of crystal orientation is a reliable method and allows the fabrication of vertical 2D heterostructures with high‐precision control of twist angle.

In conclusion, we discovered that 1D cyanide chains are oriented with threefold symmetry by “van der Waals epitaxy” on seven different hexagonal 2D crystals. The surface potential of the hexagonal 2D lattice induces the alignment of 1D cyanide chains along the zigzag lattice direction. AgCN microwires were used as rotational alignment markers, and we demonstrated fabrication of graphene/h‐BN heterostructures, aiming for a 0.0^°^ twist angle, and achieving a twist angle <0.1^°^.

## Experimental Section

*Materials Preparation*: Single‐island graphene was grown by CVD on single‐crystal Cu(111) foils,^14^ and transferred to a 300 nm SiO_2_‐on‐Si substrate. Monolayer MoSe_2_ flakes were grown by the pulse laser deposition assisted selenization method. Thin films of MoO_3_ were deposited onto a sapphire substrate by pulse laser deposition. The deposited MoO_3_ films were then converted into crystallized monolayer MoSe_2_ by the selenization process carried out in a two‐zone hot wall furnace.^15^ h‐BN, MoS_2_, MoSe_2_, MoTe_2_, WS_2_, and WSe_2_ flakes (purchased from HQ Graphene) were prepared directly on a 300 nm SiO_2_/Si substrate by mechanical exfoliation from a single crystal. AgCN microwires were grown by the drop‐cast method. The solution of AgCN (2 × 10^−3^ m) was prepared by dissolving AgCN (26.8 mg, 0.2 mmol, 99%, Sigma‐Aldrich) in ammonia solution (100 mL, 14.8 m, Samchun). A volume of 1.5 µL of the AgCN aqueous solution (2 × 10^−3^ m) was dropped onto the 2D materials either on the SiO2/Si substrate or on TEM grids using a micropipette. The substrates were subsequently dried at 75 –85 °C on a hot plate for 15 min in air. AuCN and Cu_0.5_Au_0.5_CN nanowires were prepared on various 2D crystals using the same growth method with a solution (0.8 × 10^−3^ m) obtained by dissolving AuCN (17.8 mg, 0.08 mmol, 99.99%, Alfa Aesar) and CuCN (7.1 mg, 0.08 mmol, 99%, Sigma‐Aldrich) in ammonia solution (100 mL, 14.8 m, Samchun).

*TEM Sample Preparation*: Graphene TEM grids were prepared using CVD‐synthesized graphene that was directly transferred to Quantifoil TEM grids without polymer support.^16^ For the TEM samples using other 2D crystals, 2D flakes were mechanically exfoliated onto a thin poly‐methyl‐methacrylate (PMMA)‐coated SiO_2_ (300 nm)/Si substrate. Then, a TEM grid was attached onto the substrate and 2D flakes were transferred to the TEM grid through the removal of the PMMA layer in acetone.

*Experimental Characterizations*: Optical microscope images were acquired using a Leica DM‐750M with visible light. TEM images and SAED patterns were obtained with an FEI Tecnai‐F20 operated at 200 kV and a double Cs‐corrected JEOL ARM‐200F operated at 80 kV. The electron diffraction and image simulations were performed using MacTempas software. TEM imaging acquisition conditions including a Cs value of −12 µm, convergence angle value of 0.1 mrad, mechanical vibration value of 0.5 Å, and defocus values (−2 nm for AgCN and −7 nm for Cu_0.5_Au_0.5_CN) were used for simulations. Atomic structures used for the TEM simulation were modeled using the Virtual NanoLab program. AgCN with *a* = *b* = 5.996 Å and *c* = 5.259 Å, and Cu_0.5_Au_0.5_CN with *a* = *b* = 3.396 Å and *c* = 4.931 Å were used.^5^ AFM measurements were done with a Veeco DI‐3100 AFM under ambient conditions. For the polarized Raman measurements, the 532 nm (2.33 eV) line of a diode‐pumped‐solid‐state (DPSS) laser was used as the excitation source. The laser beam was focused onto the sample by a 50× microscope objective lens (0.8 N.A.), and the scattered light was collected and collimated by the same objective. The laser power was kept below 100 µW to avoid local heating. An achromatic half‐wave plate was used to rotate the polarization of the linearly polarized laser beam to the desired direction. The analyzer angle was set such that photons with polarizations parallel to the incident polarization could pass through. Another achromatic half‐wave plate was placed in front of the spectrometer to keep the polarization direction of the signal entering the spectrometer constant with respect to the groove direction of the grating.^17^ An XPS analysis was carried out on a Thermo Fisher K‐alpha spectrometer with 100 µm beam size.

*Calculations*: The calculations for the AgCN nanowires on 2D crystals were performed using density functional theory (DFT) implemented in the Vienna Ab‐initio Simulation Package (VASP) with a projector‐augmented‐wave (PAW) method.^18^ To describe the van der Waals interaction between the AgCN chains and 2D crystals with a hexagonal lattice, the Perdew–Bruke–Enzerhof (PBE)‐D3 scheme^19^ was employed for the exchange correlation energy functional. The kinetic energy cutoff was set to 500 eV. The first Brillouin zone integration was performed using the Monkhorst–Pack scheme^20^ with 3 × 3 × 1 *k*‐point sampling for a 10 × 10 × 1 cell. To calculate the energy of the AgCN chains on 2D crystals, a chain structure of AgCN consisting of 15 atoms was used. The 2D potential energy surface of AgCN on the 2D crystals was constructed using the Lennard–Jones potential as follows

(1) $$V_{\text{LJ}}=\;\varepsilon \left[{\left(\frac{r_{0}}{r}\right)}^{12}-2{\left(\frac{r_{0}}{r}\right)}^{6}\right]$$

where ε and *r*_0_ denote the bonding energy of the chains and distance between the chains and 2D crystals corresponding to the minimum potential, respectively. The values were calculated from the DFT calculations (**Table** **1**).

**Table 1:** The values calculated from DFT calculations

	ε [eV]	*r*_0_ [Å]
Graphene	−0.037	3.0
MoS_2_	−0.177	3.0

## Conflict of Interest

The authors declare no conflict of interest.

## Supporting information

Supporting Information
