# Supplementary material for: Universal Oriented van der Waals Epitaxy of 1D Cyanide Chains on Hexagonal 2D Crystals
Source: Adv Sci (Weinh). 2019 Dec 19;7(4):1900757. doi: 10.1002/advs.201900757 (PMC7029641; doi:10.1002/advs.201900757)
Supplement: Supplementary file 1 — Supporting Information [file ADVS-7-1900757-s001.pdf]

Copyright WILEY-VCH Verlag GmbH & Co. KGaA, 69469 Weinheim, Germany, 2019.

ADVANCED SCIENCE Open Access

Supporting Information

for *Adv. Sci.,* DOI: 10.1002/advs.201900757

Universal Oriented van der Waals Epitaxy of 1D Cyanide Chains on Hexagonal 2D Crystals

Yangjin Lee, Jahyun Koo, Sol Lee, Jun-Yeong Yoon, Kangwon Kim, Myeongjin Jang, Jeongsu Jang, Jeongheon Choe, Bao-Wen Li, Chinh Tam Le, Farman Ullah, Yong Soo Kim, Jun Yeon Hwang, Won Chul Lee, Rodney S. Ruoff, Hyeonsik Cheong, Jinwoo Cheon, Hoonkyung Lee, and Kwanpyo Kim**

Copyright WILEY-VCH Verlag GmbH & Co. KGaA, 69469 Weinheim, Germany, 2019.

Supporting Information

Universal Oriented van der Waals Epitaxy of 1D Cyanide Chains on Hexagonal 2D Crystals

Yangjin Lee[1,2], Jahyun Koo[3], Sol Lee[1,2], Jun-Yeong Yoon[1,2], Kangwon Kim[4], Myeongjin Jang[1], Jeongsu Jang[1,5], Jeongheon Choe[1], Bao-Wen Li[6], Chinh Tam Le[7], Farman Ullah[7], Yong Soo Kim[7], Jun Yeon Hwang[8], Won Chul Lee[9], Rodney S. Ruoff[6,10,11,12], Hyeonsik Cheong[4], Jinwoo Cheon[2,13,14], Hoonkyung Lee[3,], and Kwanpyo Kim[1,2,*]*

[1]Department of Physics, Yonsei University, Seoul 03722, Korea.

[2]Center for Nanomedicine, Institute for Basic Science (IBS), Seoul 03722, Korea.

[3]Department of Physics, Konkuk University, Seoul 05029, Korea.

[4]Department of Physics, Sogang University, Seoul 04107, Korea.

[5]Department of Physics, Ulsan National Institute of Science and Technology (UNIST), Ulsan 44919, Korea.

[6]Center for Multidimensional Carbon Materials (CMCM), Institute for Basic Science (IBS), Ulsan 44919, Korea.

[7]Department of Physics, University of Ulsan, Ulsan 44610, Korea.

[8]Institute of Advanced Composite Materials, Korea Institute of Science and Technology (KIST), Jeonbuk 55324, Korea.

[9]Department of Mechanical Engineering, Hanyang University, Ansan 15588, Korea.

[10]Department of Chemistry, Ulsan National Institute of Science and Technology (UNIST), Ulsan 44919, Korea.

[11]School of Materials Science and Engineering, Ulsan National Institute of Science and Technology (UNIST), Ulsan 44919, Korea.

[12]School of Energy and Chemical Engineering, Ulsan National Institute of Science and Technology (UNIST), Ulsan 44919, Korea.

[13]Department of Chemistry, Yonsei University, Seoul 03722, Korea

[14]Graduate Program of Nano Biomedical Engineering, Yonsei-IBS Institute, Yonsei University, Seoul 03722, Korea

*Address correspondence to H.L. (hkiee3@konkuk.ac.kr) and K.K. (kpkim@yonsei.ac.kr)

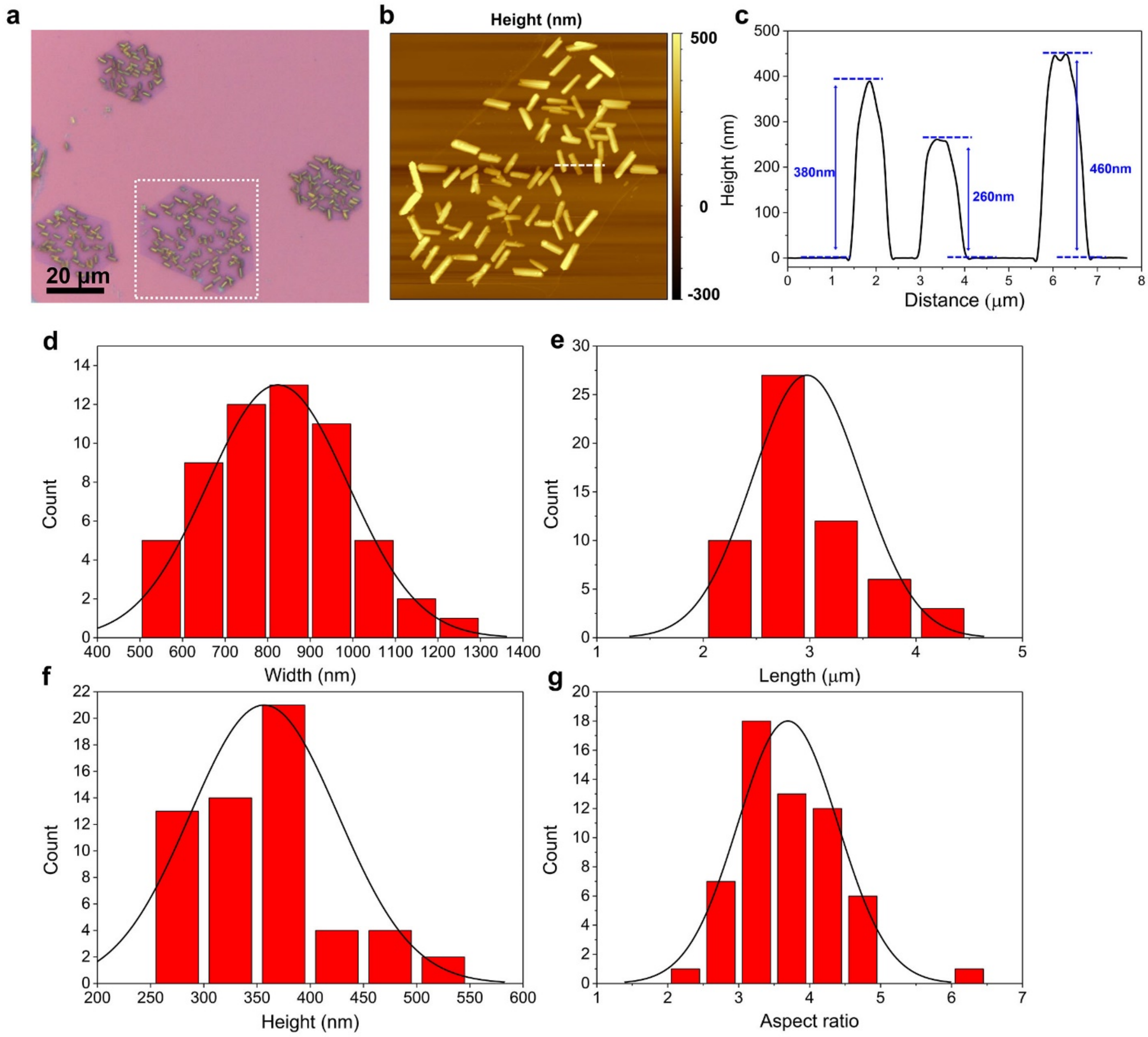

Figure S1. The dimensional distribution of AgCN wires grown on CVD single-crystal aligned graphene. (a) Optical micrograph of AgCN wires on graphene. (b) AFM height image of AgCN wires on graphene. (c) Height plot from figure (b) white dashed line. (d-g) The dimensional distribution of AgCN wires grown on graphene. (d) The width, (e) length, (f) height, and (g) aspect ratio (length/width).

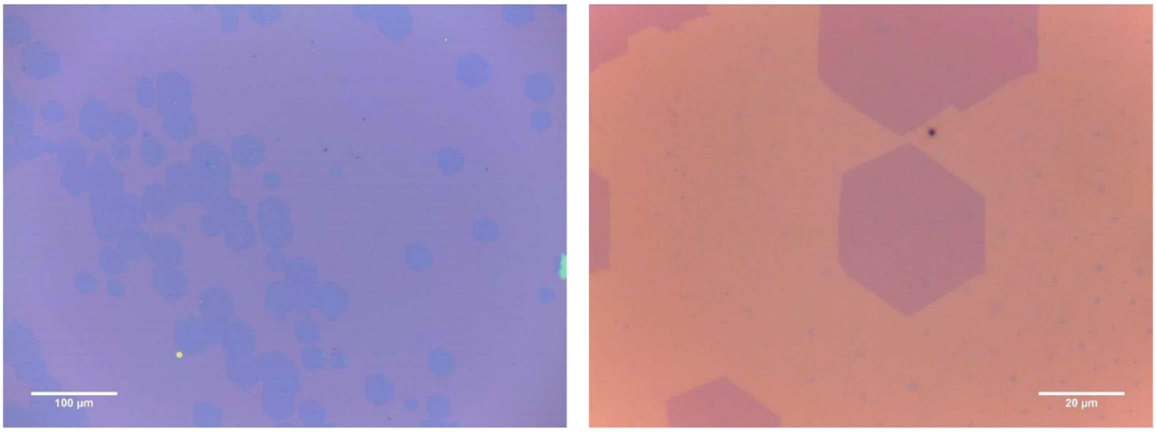

Figure S2. Aligned Single-Crystal Graphene Domains. Graphene islands transferred to SiO_2/Si substrates. Each domain is single-crystalline and the crystal orientation between islands is aligned. The edge of graphene islands is along the zigzag lattice direction of graphene.

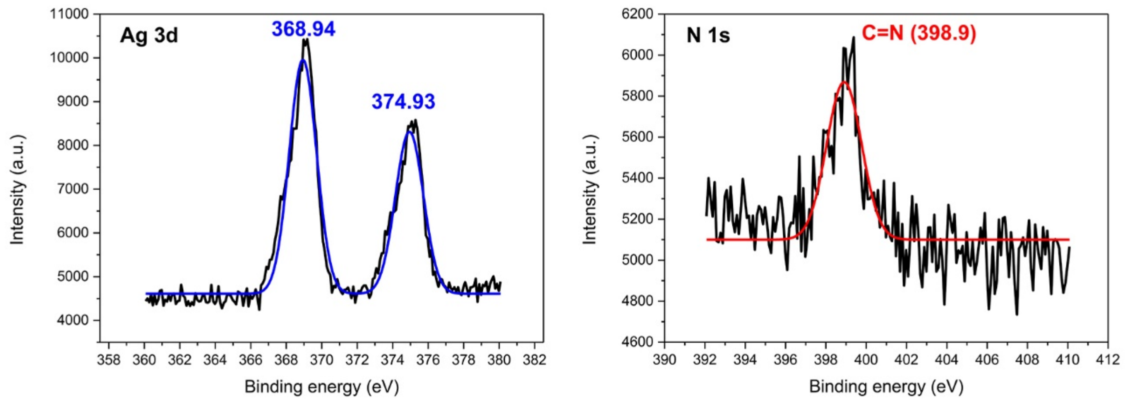

Figure S3. X-ray photoemission spectra of AgCN crystals. Ag 3d (left) and N 1s (right) spectra.

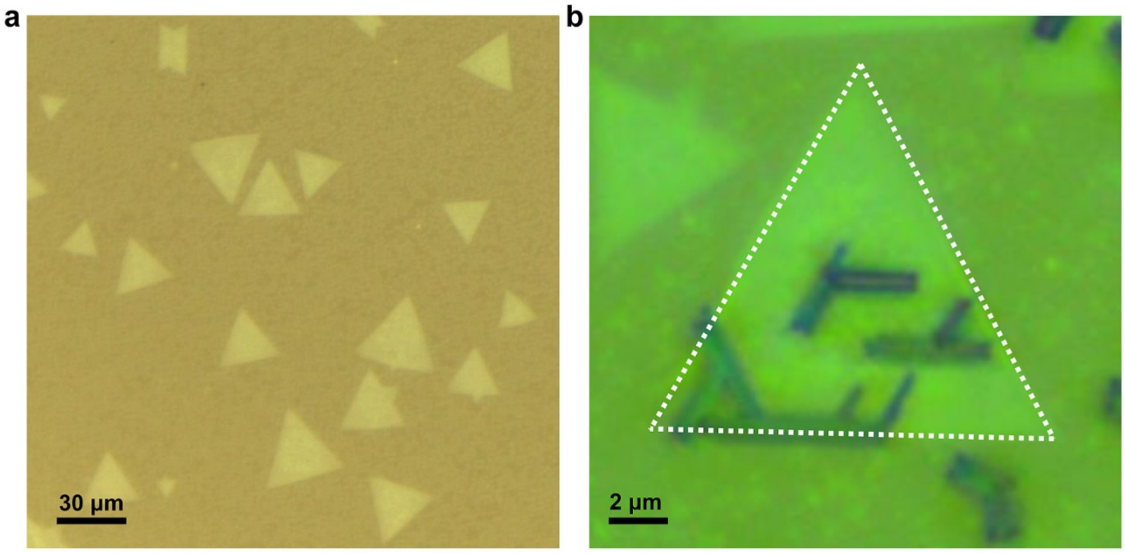

Figure S4. Optical microscope image of $MoSe_2$ flakes grown on sapphire substrate (a) before and (b) after the growth of AgCN microwires.

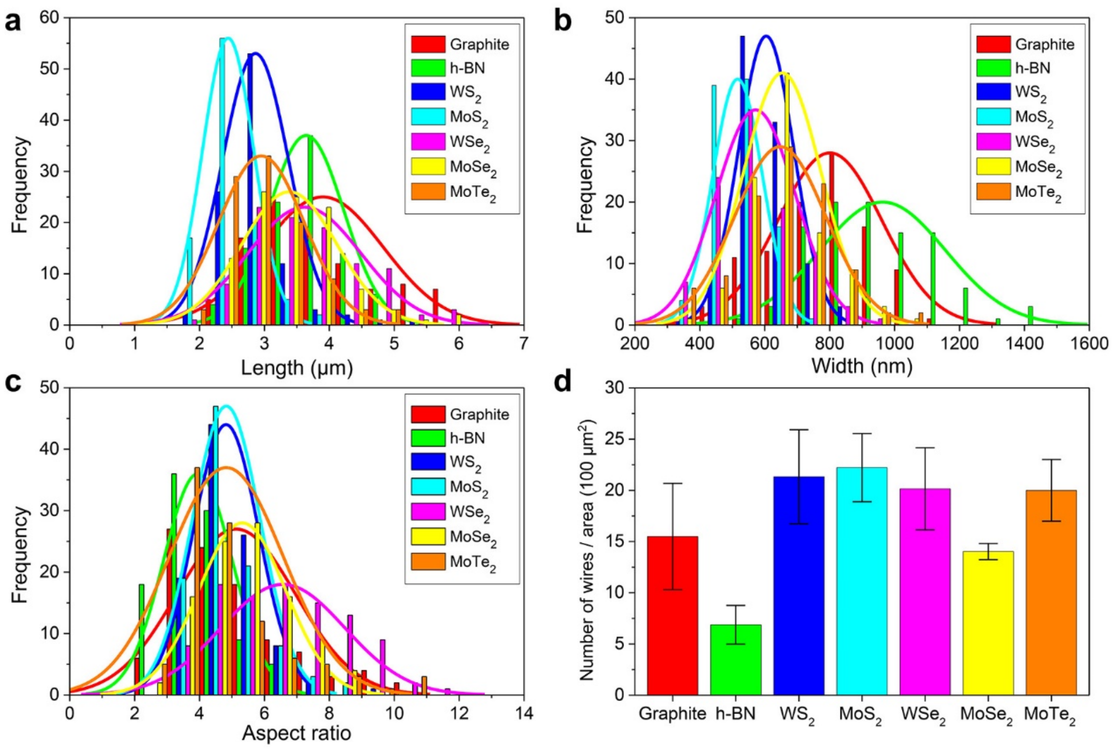

Figure S5. The dimensional distribution of AgCN wires grown on various hexagonal 2D crystals. (a-d) The dimensional distribution of AgCN wires grown on hexagonal 2D crystals. (a) The length, (b) width, (c) aspect ratio (length/width), and (d) density (number of wires/area). Optical microscope images were used to analyze the dimensional distribution.

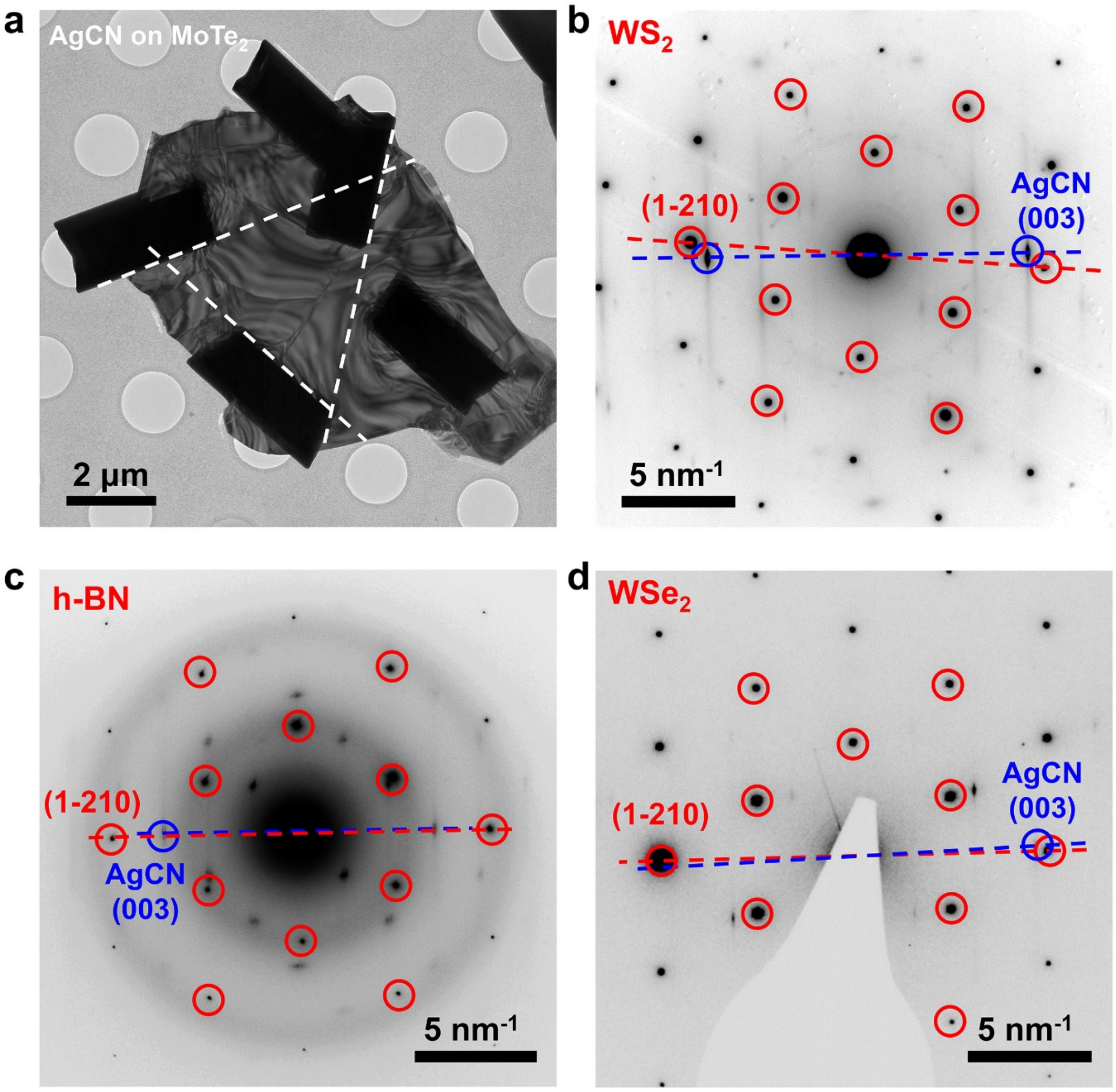

Figure S6. TEM image and SAED of AgCN crystals on various 2D crystals. (a) TEM image of AgCN wires on $MoTe_2$. (b-d) SAED of AgCN wires on (b) WS_2, (c) h-BN, and (d) WSe_2.

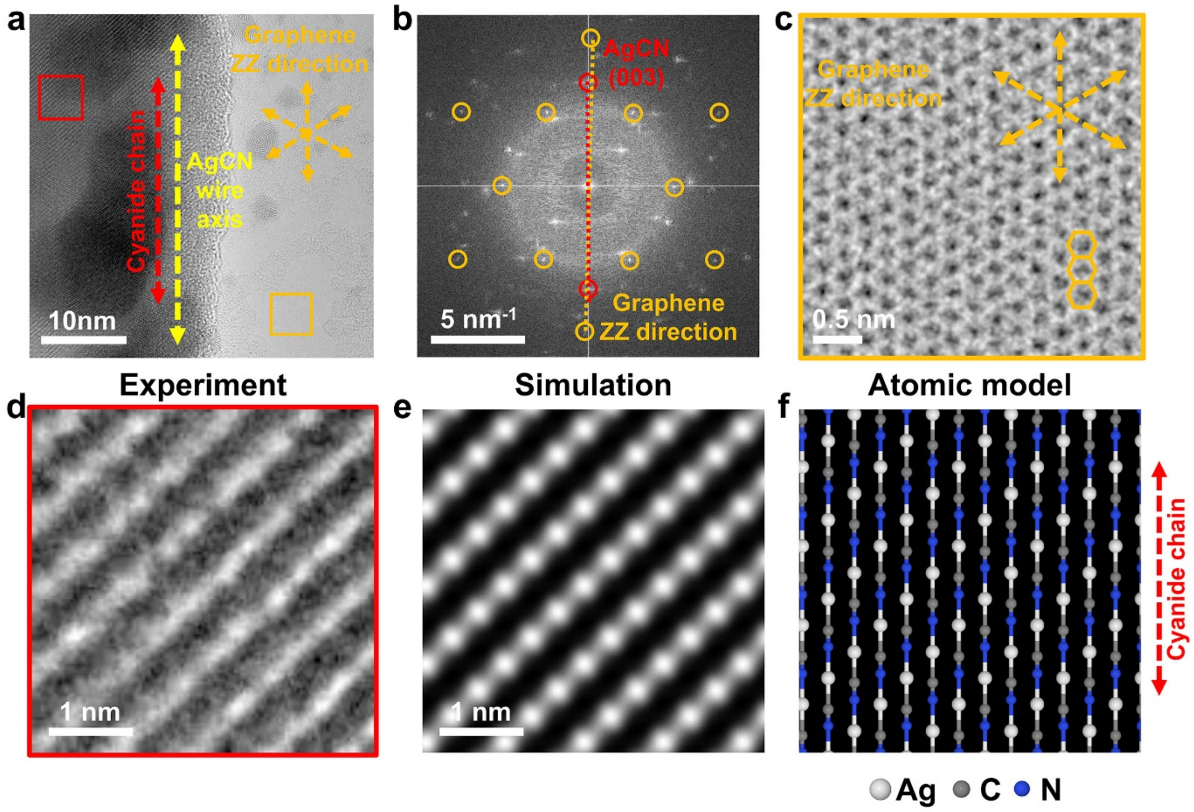

Figure S7. Atomic-scale TEM imaging of oriented alignment of AgCN wires on graphene. (a) TEM image of a AgCN wire on graphene. Orange- and red-colored boxes are the field of view for panel c and d. (b) Fourier transform of panel (a). Diffraction signal from graphene and AgCN chains are marked. (c) High-resolution TEM image of graphene substrate. (d) High-resolution TEM image of AgCN chains. (e) TEM image simulation using the crystal structure shown in panel f. (f) Atomic model of AgCN chains.

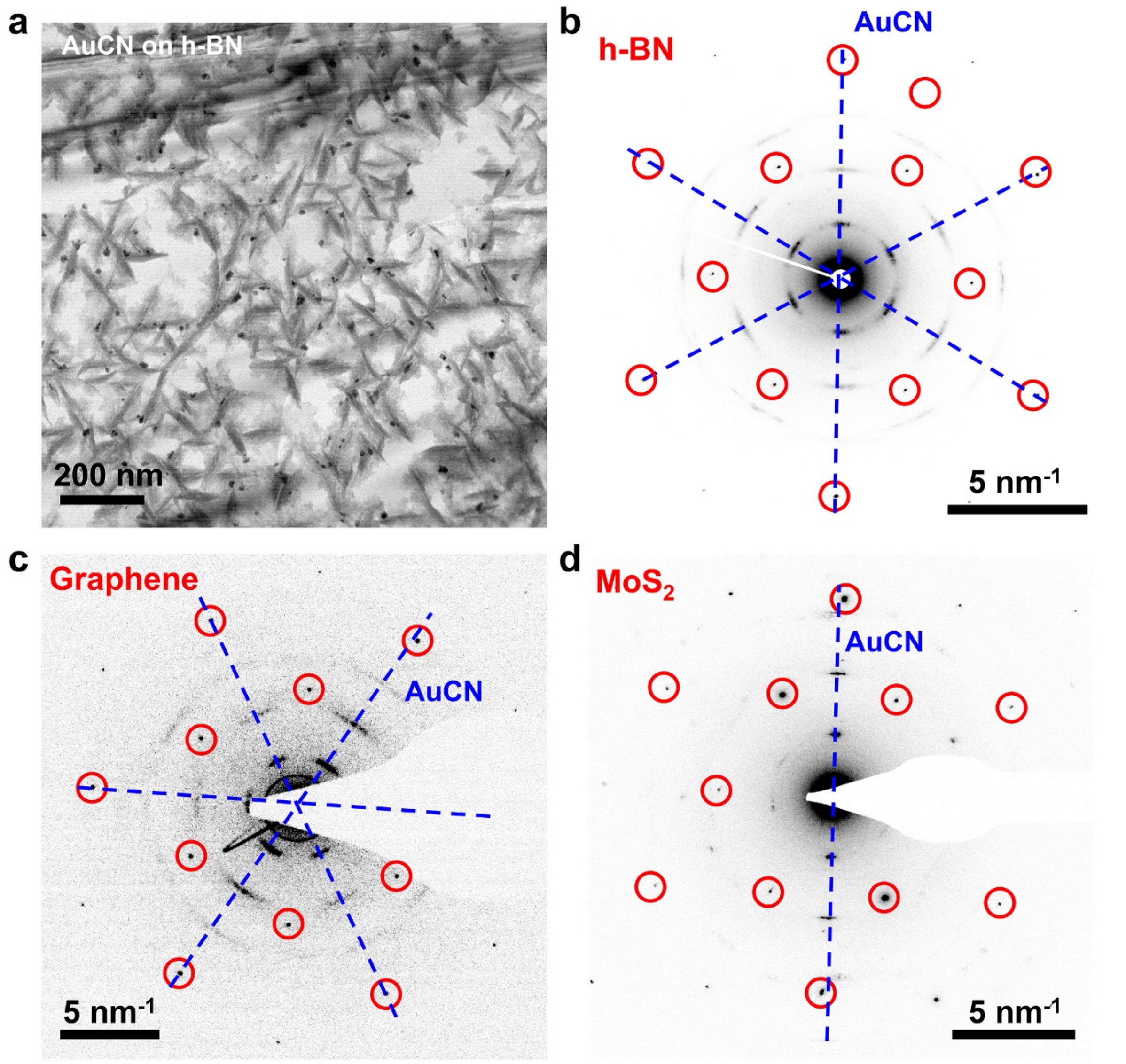

Figure S8. TEM images and SAED of AuCN nanowires assembled on graphene, h-BN, and MoS_2. (a) TEM image of AuCN nanowires on h-BN. (b-d) SAED of AuCN nanowires on (b) h-BN, (c) graphene, and (d) MoS_2.

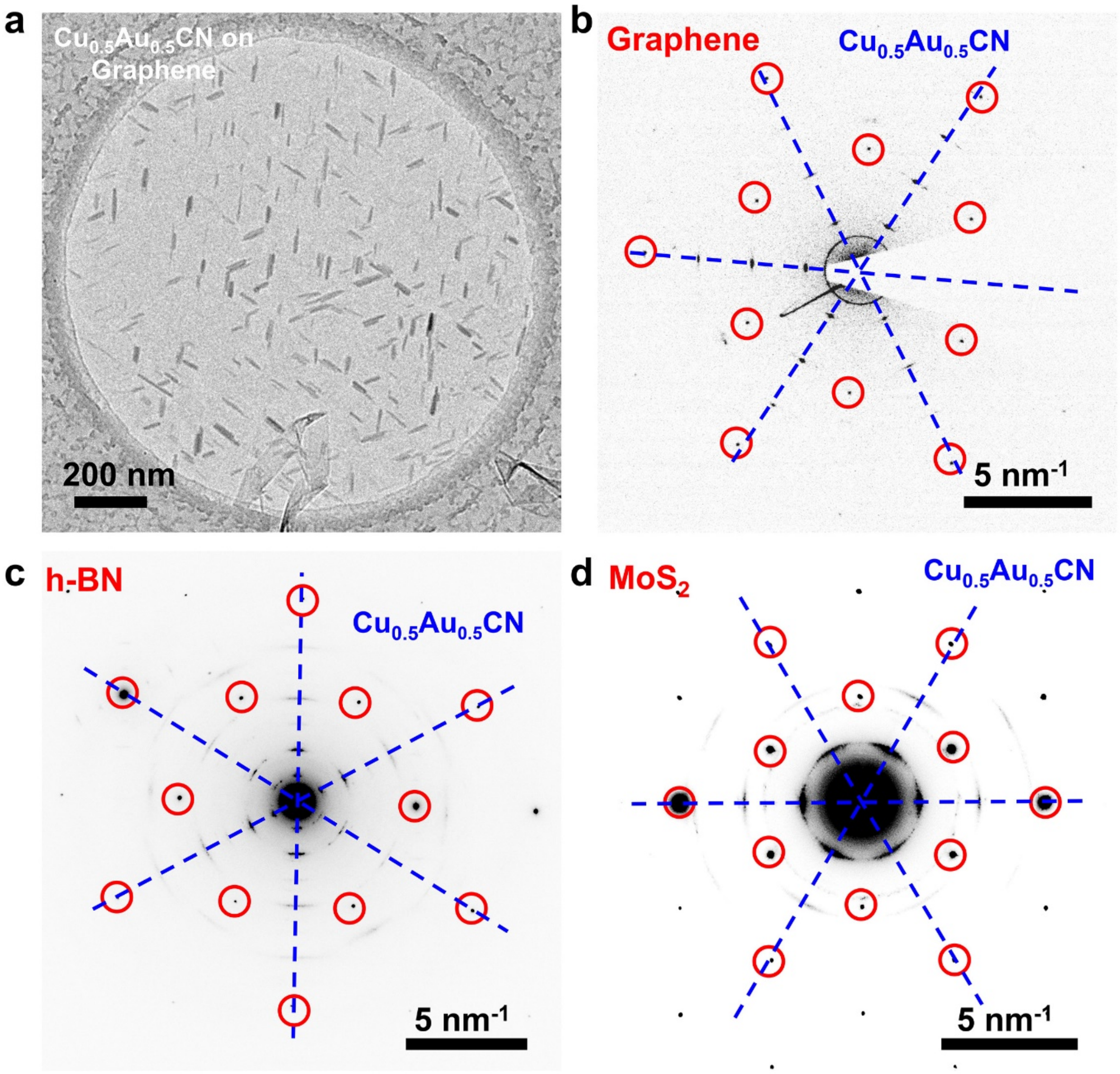

Figure S9. TEM images and SAED of $Cu_{0.5}Au_{0.5}CN$ nanowires assembled on graphene and other 2D crystals. (a) TEM image of $Cu_{0.5}Au_{0.5}CN$ wires on graphene. (b-d) SAED of $Cu_{0.5}Au_{0.5}CN$ wires on (b) graphene, (c) h-BN, and (d) MoS_2.

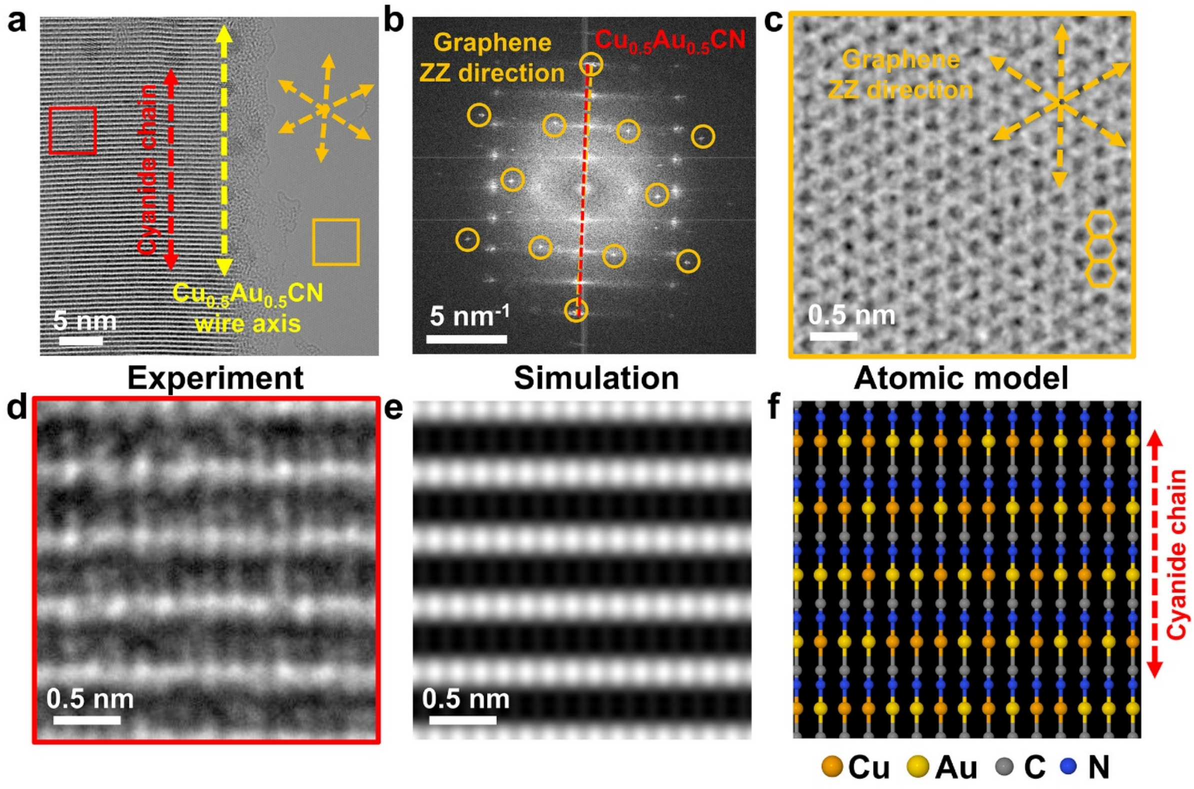

Figure S10. Atomic-scale TEM imaging of oriented alignment $Cu_{0.5}Au_{0.5}CN$ wires on graphene. (a) TEM image of $Cu_{0.5}Au_{0.5}CN$ wire on graphene. Orange- and red-colored boxes are the field of view for panel c and d. (b) Fourier transform of panel (a). Diffraction signal from graphene and $Cu_{0.5}Au_{0.5}CN$ chains are marked. (c) High-resolution TEM image of graphene substrate. (d) High-resolution TEM image of $Cu_{0.5}Au_{0.5}CN$ chains. (e) TEM image simulation using the crystal structure shown in panel f. (f) Atomic model of $Cu_{0.5}Au_{0.5}CN$ chains.

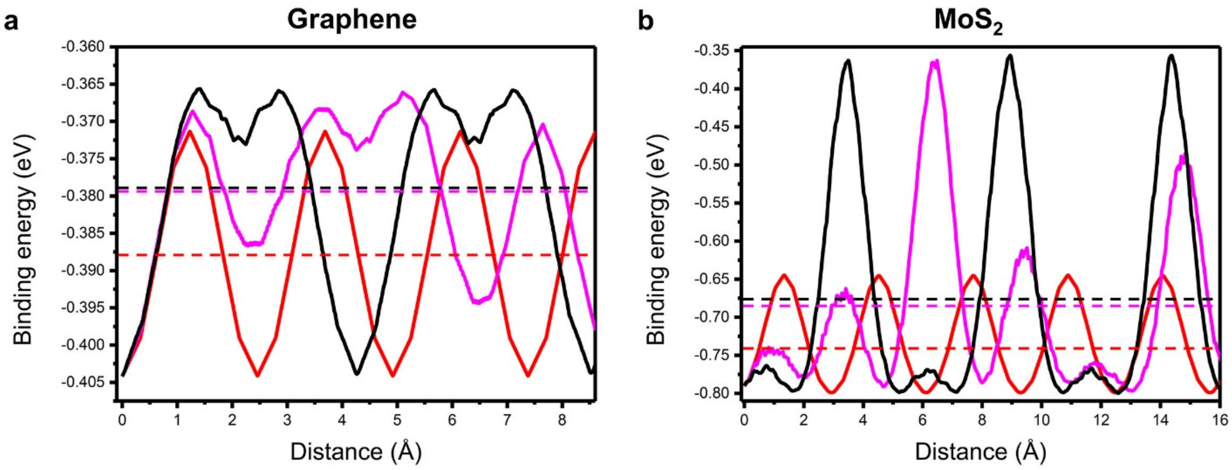

Figure S11. The potential energy variation of an AgCN wire on graphene and MoS_2. (a) The potential energy of an AgCN wire on graphene along a zigzag direction (red line), an armchair direction (black line), and rotated 15 degrees from the zigzag lattice direction (magenta). (b) The potential energy of an AgCN wire on MoS_2 along a zigzag direction (red line), an armchair direction (black line), and rotated 15 degrees from the zigzag lattice direction (magenta). Dashed dot lines indicate the average potential energy.

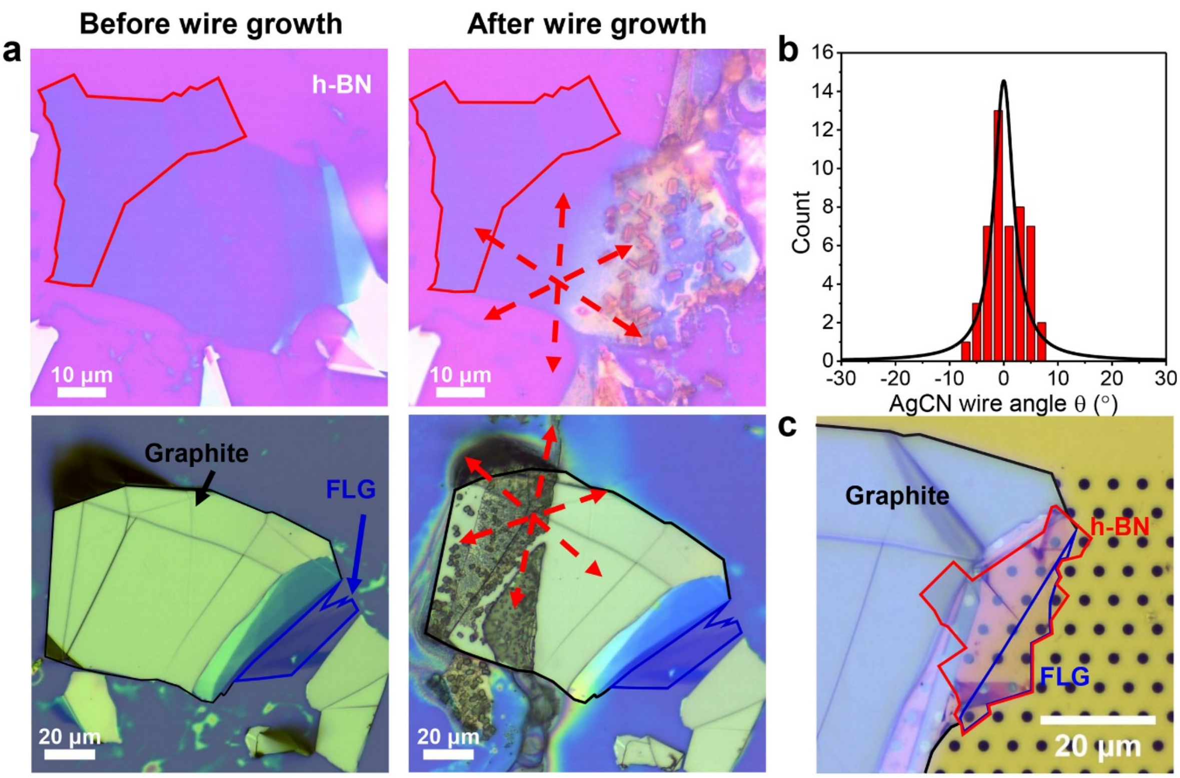

Figure S12. Fabricated graphene/h-BN heterostructure using AgCN wire markers. (a) Before (left) and after (right) AgCN wire growth on h-BN (top) and graphene (bottom). The half of flake surface was protected by PDMS to maintain the pristine surface. The red arrows indicate the zigzag lattice directions of h-BN and graphene. (b) Angular histogram of AgCN wire axis on the h-BN flake. (c) Optical microscope images of fabricated graphene/h-BN heterostructure on a holey Si_3N_4 TEM grid.

Table S1. Lattice parameters of 2D crystals investigated in this work. (Units: Å)

	A (Zigzag)	B (Armchair)
Graphene	2.46	4.26
h-BN	2.50	4.33
WS_2	3.15	5.46
MoS_2	3.16	5.47
WSe_2	3.28	5.68
$MoSe_2$	3.29	5.70
$MoTe_2$	3.52	6.10

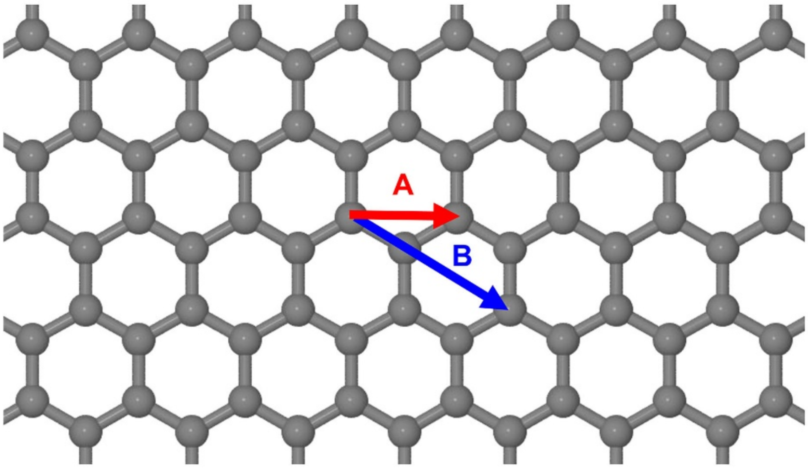

Table S2. Lattice parameters of chained cyanides studied in this work.

	AgCN	AuCN	$Cu_{0.5}Au_{0.5}CN$
a/Å	6.00 (6.007)*	3.31 (3.393)*	3.40 (3.396)*
c/Å	5.23 (5.259)*	5.03 (5.066)*	4.93 (4.931)*

* From reference [7]

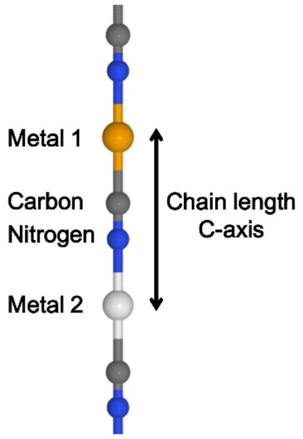

Supporting Note 1

Measurement of lattice mismatch between graphene and h-BN

The set of graphene diffraction peaks {0-220} were calibrated to be 1.065 Å in diffraction images and TEM images. After the calibration process, the h-BN lattice constant and the lattice mismatch were experimentally measured. The lattice mismatch δ between graphene and h-BN was measured to be 1.76 ($\pm$0.01) % and the h-BN lattice constant was measured to be 2.504 Å.

Theoretical moiré distance (λ) was calculated using the following equation

$$\lambda = \frac{(1+\delta)a}{\sqrt{2(1+\delta)(1-cos\theta)+\delta^2}}$$

where a = 2.460 Å is the graphene lattice constant, θ is the relative rotation angle between graphene and h-BN, and δ is lattice mismatch between graphene and h-BN.
